# Global Initiative for Children’s Surgery (GICS) Pediatric Trauma Care Initiative: A Call for a Comprehensive Approach to a Global Problem

**DOI:** 10.3390/children11060666

**Published:** 2024-05-29

**Authors:** Abdelbasit E. Ali, Adesoji Ademuyiwa, Simone Abib, Charles Carapinha, Fazal Nouman Wahid, Udo Rolle, Kokila Lakhoo

**Affiliations:** 1 Department of Pediatric Surgery, King Saud Medical City, Imam Abdelaziz bin Mohamed bin Saud Street, Olayshah, Riyadh 12746, Saudi Arabia; f.wahid@ksmc.med.sa; 2 Department of Surgery, College of Medicine, University of Lagos, Paediatric Surgery Unit, Lagos University Teaching Hospital, Lagos Nigeria 102216, Nigeria; aademuyiwa@unilag.edu.ng; 3 Pediatric Surgery, Federal University of São Paulo, São Paulo 04023-062, Brazil; simoneabib@uol.com.br; 4 Netcare Alberton Hospital, Johannesburg 1449, South Africa; doc@paediatricsurgeon.cc; 5 Department of Pediatric Surgery and Pediatric Urology, University Hospital Frankfurt/M, 60590 Frankfurt, Germany; rolle@med.uni-frankfurt.de; 6 Nuffield Department of Surgical Sciences, Paediatric Surgery, University of Oxford, Oxford University Hospitals, Oxford OX3 9DU, UK; kokila.lakhoo@paediatrics.ox.ac.uk

**Keywords:** global children’s surgery, trauma, specialization

## Abstract

Introduction: Trauma is a major problem which has a significant health, social, and economic impact. Particularly, pediatric trauma carries substantial mortality and morbidity. This is a great concern for subspecialized general and pediatric surgeons. Therefore, a global initiative for pediatric trauma care is warranted and should be initiated. Aim: The international association “Global Initiative for Children’s Surgery” (GICS) would like to propose and organize a children’s trauma care (CTC) initiative. This initiative should comprehensively address pediatric trauma management globally, especially in low- and middle-income countries (LMICs). The initiative seeks to achieve a structured cooperation and collaboration with respective sister organizations and local stakeholders. Methods: The initiative will address these relevant aspects: 1. first aid; 2. prehospital primary trauma care; 3. hospital primary trauma care; 4. advanced care (ATLS); 5. diagnostic facilities; 6. operation room (OR) equipment; 7. specialized surgical services; 8. rehabilitation; 9. registry, research, and auditing; 10. specialization in pediatric trauma; 11. capacity and confidence building in pediatric trauma; 12. prevention. The GICS CTC provided activities have been recorded and evaluated in a structured manner. This statement paper is based on data of a narrative review as well as expert opinions. Results: The Trauma Working Group of GICS provided specialized trauma prevention leaflets available for translation to different languages. A one-day children’s primary trauma course has been designed to be delivered at the physical GICS meetings. Exercising advocacy, the group addressed several meetings on prevention of pediatric trauma, which included the 75th United Nations General Assembly (UNGA) (2020), GICS IVth meeting in Johannesburg (2020), Norwich (UK) Joint SPRINT Symposium on Pediatric Surgery for Pediatricians (2021), the second online Pan African Pediatric Surgical Association (PAPSA) meeting (2021), the seventh World Congress of the World Federation of Associations of Pediatric Surgeons (WOFAPS) in Prague (2022), and GICS pediatric trauma webinar (2023). Additionally, the working group participated in the preparations of a pediatric trauma module for the World Health Organization (WHO) and published several related studies. The contents of the selected articles added relevant information to the categories stated above. Conclusions: The CTC initiative of GICS is proposed as a mean to address pediatric trauma comprehensively through a process of collaboration and advocacy with existing organizations to achieve awareness, health education, prevention, health, and training. Further, it will support the provision of suitable facilities to health institutions. The establishment of a specialization in pediatric trauma is encouraged. GICS CTC initiative aims to improve pediatric trauma care in LMICs by developing injury prevention strategies; optimizing the use of locally available resources; obtaining commitment by LMICs governments; improvement in all fields of hospital care; improvements in infrastructure, education and training, and attention to data registry and research.

## 1. Introduction

Trauma is a common problem globally with profound health and socioeconomic impacts. Pediatric trauma is associated with high morbidity and mortality, especially in LMICs. Trauma inclusion as a separate entity within the Global Alliance for Surgery, Obstetric, Trauma, and Anesthesia Care (The G4 Alliance) [1] signifies attention to the importance of this still neglected health threat in many parts of the world. In view of its association with significant mortality, disability, and socioeconomic implications, trauma remains a genuine concern to general and subspecialized pediatric surgeons.

### 1.1. Justification of GICS Children Trauma Care (CTC) Initiative

Trauma is a major cause of fatalities overall. There is higher trauma-related mortality compared to malaria, tuberculosis, and HIV/AIDS. It is estimated that injuries cause 40% of all deaths in children 1–14 years of age, and furthermore 30% of disability with high economic cost and psycho-social impact [2]. LMICs have the highest rate of childhood injury related deaths at 95% [3,4]. The overall mortality of life-threatening but treatable injuries is 36% in LMICs compared to 6% in high income countries (HICs) [5].

While trauma is universal, non-discriminating, disturbing, distressing, disfiguring, disabling, and can be fatal, much can be done to reduce morbidity and mortality of this entity particularly in the pediatric population. This includes prevention, prehospital and in-hospital care, follow-up care, to rehabilitation.

Financially, injuries are a leading cause of enormous costs. Estimated costs of preventable trauma are US$ 1.3 trillion in Europe and North America [6]. Remarkable progress in injury prevention and injury treatment resulted from the implementation of trauma systems in the USA in the 1960s. Subsequently, the mortality rate of pediatric trauma dropped by 50% during the 1980s and 1990s in the USA. The overall mortality from unintentional trauma in children decreased from 15 per 100,000 to 8.3 per 100,000 in 2013 [7,8].

The maternal and infant mortality rates in Africa are among the highest in the world. On the other hand, the average annual per capita income is $400 or less. Furthermore, the expenditure for health is less than $14 per capita per annum [9] in most sub-Saharan African countries. This clearly falls short of meeting the basic requirements for healthcare, including trauma.

Major problems in the management of injured patients in LMICs result from often required transfers over long distances and the lack of adequate ambulance facilities, which leads to late arrival after injury [10]. There are small numbers of health professionals with limited training. Further, there is a lack of necessary subspecialty services such as orthopedics, neurosurgery, and rehabilitation. Additionally, there may be no high-tech equipment (and sometimes lack of fundamental requirements such as electricity, oxygen, and running water).

To minimize the negative effects, good standards of pre- and in-hospital trauma care are required around the world, particularly in LMICs.

Provision of fundamental and essential requirements for an adequate, comprehensive pediatric trauma service, within a pediatric-friendly environment, taking into consideration their anatomical, physiological, and psychological peculiarities, is a genuine and urgent necessity. Consequently, system development and capacity building are necessary for appropriate children’s trauma care.

### 1.2. Aim

Global Initiative for Children’s Surgery (GICS) has proposed a children’s trauma care (CTC) initiative aiming to address pediatric trauma management comprehensively, globally, and in LMICs.

## 2. Methods

To achieve a global coverage, GICS CTC seeks to provide a comprehensive practical approach designed to address all the following aspects:

1. first aid; 2. prehospital primary trauma care; 3. hospital primary trauma care; 4. advanced care (ATLS); 5. diagnostic facilities; 6. operation room (OR) equipment; 7. specialized surgical services; 8. rehabilitation; 9. registry, research, and auditing; 10. specialization in pediatric trauma; 11. capacity and confidence building in pediatric trauma; 12. prevention.

This should be achieved through advocacy, including utilization of platforms, webinars, publications, preparation of educational material, collaboration, and cooperation with other concerned organizations, in addition to invitation of local healthcare providers to implement a trauma program.

The GICS CTC provided activities have been recorded and evaluated in a structured manner.

This statement paper is based on data of a narrative review as well as expert opinions. Available data were searched for all above mentioned major aspects in PubMed, Google search engines using the search terms pediatric trauma, children, injuries, first aid, trauma care, rehabilitation, LMICs, prehospital care, and trauma registry.

The articles were filtered and selected according to their relevance to the topics of interest.

## 3. Results

GICS focuses on advocacy and works in collaboration with existing societies to facilitate and ensure that children in LMICs can have access and benefit from respective resources that include educational material, curricula, guidelines, training courses, workshops, tools, instruments, equipment, activities, and events. Some of the material has been replicated using locally available facilities. The GICS Trauma Working Group has produced simplified trauma prevention leaflets available for translation to different languages which are uploaded in its website. A one-day children’s primary trauma course was prepared to be conducted at the GICS physical meeting. Exercising advocacy, the group addressed the following meetings: GICS IVth meeting in Johannesburg (January 2020), the 75th United Nations General Assembly (UNGA) in September 2020, Norwich (UK) Joint SPRINT Symposium on Pediatric Surgery for Pediatricians (2021), the second online Pan African Pediatric Surgical Association (PAPSA) meeting (November 2021), the seventh World Congress of the World Federation of Associations of Pediatric Surgeons (WOFAPS) in Prague (October 2022), and organized a pediatric trauma awareness webinar in February 2023. Additionally, GICS CTC has participated in the preparation of a module for WHO and has published its results including a joint paper on surgery in the first 20 years of life [11], which concluded that improving surgical service capacities at first-level hospitals in LMICs has the potential to avert many deaths within the first 20 years of life. A publication from Uganda showed that after completion of a train-the-trainer trauma course the short-term trauma training interventions were influenced positively, but long-term impact was limited due to barriers to adopting best practices [12].

Articles reviewed based on the above-mentioned search terms were 53. The final number of articles included was 36, which fulfilled our search criteria and were relevant to the topic. The rest were excluded either due to deficiencies with no full text available, repetition of messages in the selected articles or case reports.

Contents of the selected articles will be mentioned in the aspects/categories below.

### 3.1. GICS CTC Initiative and Recommendations for Improvements to Trauma Care in LMICs

GICS trauma care initiative advocates an approach that covers all necessary components of trauma care. Here is an account of the importance and justification of each of the aspects to be addressed:

#### 3.1.1. First Aid

Public education on principles of first aid in addition to provision of training and required equipment/box, material to guide witnesses and attendants’ response to trauma to make a difference in the outcome.

A recent systematic review which covered 10 studies worldwide revealed that the frequency of first aid applied to trauma casualties ranged from 10.7% to 65%, whereas wrong first aid measures have been undertaken in up to 83.7% of the cases [13].

Another recent study showed that instructing school children to teach cardiopulmonary resuscitation (CPR) to their friends and relatives leads to positive attitude towards CPR [14].

#### 3.1.2. Prehospital Primary Trauma Care

Primary trauma care training through courses by the Primary Trauma Care Foundation, utilizing locally available resources, enables healthcare personnel and community individuals and groups (relatives/neighbors, drivers, teachers, public, paramedics) to perform better and provide safe and effective care to trauma patients within the precious time that follows the trauma event. The course has been conducted in 84 countries, and each year 160 courses are delivered, with >100,000 doctors trained since its inception in 1996 [15].

The methods of transportation for trauma patients in various parts of the world include ambulance, own car, taxi, bus, motorcycle, animal pulled cart, carrying by hands and walking assisted, with variable risks of complicating the already inflicted injury. Therefore, ambulance service improvement is a necessity for safe transportation, timely resuscitation, and avoidance of compounding trauma-related injuries. Assessing available reports on the respective impact of training on mortality and morbidity showed the following. The training of non-specialists to perform primary treatment of open fractures and applying external fixation led to a decrease in the amputation rates from 100% to 21% over 7 years at the Médecins Sans Frontiers run first-level referral hospital in Masisi, Democratic Republic of the Congo [16]. A surgical skills training program for nondoctors showed substantial reductions in postoperative infection rates and trauma mortality in Cambodia [17].

Further, prehospital care system utilizing paramedics and lay-persons in rural Iraq led to increased acceptance and use of the system [18], and further showed a significant reduction in mortality. This intervention also led to an improvement in severity scores [19].

#### 3.1.3. Hospital Primary Trauma Care

Substantial progress is made in the way of improving the quality of trauma management and service provided through systematic application of advanced trauma life support (ATLS) principles at the scene or at the reception and resuscitation area in the emergency room. Utilization of charts (height, weight, percentiles,) and advanced resuscitation equipment facilitate effective resuscitation (see list of emergency tray essential equipment for an emergency room below) (Table 1) [20].

#### 3.1.4. Advanced Care (Advanced Trauma Life Support—ATLS) [21]

While most fatalities take place at the scene, there is an estimated in-hospital mortality of 1–2% [2]. It is, therefore, a necessity for the establishment of such health facilities to ensure a longitudinal program of collaboration for maintenance and training for the continuity and sustainability of the desired adequate service. Lack of facilities represent an obstacle and jeopardize patients’ care.

Therefore, required facilities include the following:

#### 3.1.5. Diagnostic Facilities (Radiology)

To sufficiently diagnose injuries, imaging modalities such as plain X-ray and ultrasound are of utmost importance. Further, advanced methods such as computerized tomography (CT) scan and magnetic resonance imaging (MRI) are utilized to determine the grade and extent of the injuries further.

The two main diagnostic imaging modalities in the developing world used for trauma/emergency services remain X-ray and ultrasound. It has been shown that these two modalities are able to meet more than 90% of the acute imaging needs in acute injuries. Especially, ultrasound is a valid tool to screen for significant trauma of the abdomen and thorax. It enables the diagnosis of i.e., abdominal organ injuries, pneumothorax, and pericardial tamponade [22].

If X-ray and ultrasound are not available, patients need to be transported to the respective facilities. This usually leads to a significant delay of the required treatment as well as increased morbidity and mortality. In the example of Nepal, one group of patients needed to travel over 10 h, others over 2 days, to reach an X-ray facility; transportation costs alone for this reason may exceed an average monthly wage [22].

The same study [22] reported on the huge shortage of imaging equipment in low- and middle-income countries in general (LMICs). There is less than 1 CT scanner per million inhabitants in LMICs compared to almost 40 scanners per million inhabitants in high-income countries (HICs). The gap is even wider for MRI and nuclear medicine equipment.

#### 3.1.6. Operation Room (OR) Facilities (Equipment)

Functional OR facilities, equipped with various but essential pieces of equipment are crucial for trauma care. This includes equipment for anaesthesia and monitoring, operating tables, light, warmers, suction, electrocautery, and C-arm X-ray machine. Modern OR’s are equipped with hybrid facilities to accommodate standard and interventional procedures. In contrast, lack of essential, basic requirements (such as flooring, walling, ventilation, cleanliness, sterilization, adequate surgical equipment and instruments, safety) is commonly observed in many LMICs, consequently and adversely affecting the quality of service. It is important to point out the encouragingly great charity initiatives such as KIDSOR (https://www.kidsor.org), which installs and provides well equipped OR’s where needed.

#### 3.1.7. Specialized Surgical Services Care (Pediatric Intensive Care Unit (PICU)

A proper trauma service cannot be conducted without the availability of intensive care unit (ICU) facilities, which significantly reduce the trauma related mortality. Specialized PICU facilities require space, equipment, and highly trained medical, nursing, and technical staff. There is a marked deficiency of PICU services in LMICs which further jeopardizes the chances for survival following major trauma in LMICs.

There is a clear lack of designated PICUs in most LMICs together with a low number of trained nursing staff, no adequate nurse to patient ratio, and lack of equipment and monitoring capability, as well as ancillary support [23]. PICUs in LMICs are usually staffed by general pediatricians, which does not always allow for specialized treatment of injuries. Available data show better survival of pediatric trauma patients in regions with an appropriate number of PICU beds [24].

There is a defined need for allied health specialties to achieve a reasonably adequate pediatric trauma service, including anaesthesia, blood bank, clinical pharmacy, laboratories, physiotherapy, specialists in nutrition, education, rehabilitation, registry and documentation for logistics, demand management, audit, KPI setting, research, coordination, development, and capacity building.

#### 3.1.8. Rehabilitation

Specialized rehabilitation together with structured follow-up is a required tool to assist the injured child, as otherwise disability could occur, and further, achievement and maintenance of optimal functioning in interaction of the injured child with their environment is secured. It has been shown that early commencement of rehabilitation leads to prevention of disability and reduced healthcare costs. Trained professionals (prosthetic, orthotic technicians, occupational therapists, physical therapists) are required for the adequate provision of assistant devices. However, even further and more complex changes in the patient’s environment are required too i.e., facilitating accessibility of a wheelchair to a school.

Ameratunga et al. showed that only 3% of individuals who need rehabilitation globally receive any kind of support. Of the 114 countries providing data to the global survey on government action in 2005, 43 countries (38%) provided no support for children with disabilities regarding assistive devices and support services [25].

Only 58.5% of all referral hospitals had rehabilitation units in LMICs, such as Zimbabwe, Zambia, Uganda, Tanzania, Rwanda, Mozambique, Malawi, Kenya, Ethiopia, and Burundi. This is even less provided in district hospitals (35.7%) [10].

A WHO compendium of “success stories” [26,27] containing two reports described the possible impact of rehabilitation interventions. A trauma center in Brazil started with a small multidisciplinary rehabilitation, which provided early rehabilitation on the trauma wards. This led to improvements in mobility and enhanced self-care. Further, the use of adaptive devices, as well as the coordination of patient follow-up with outpatient rehabilitation was introduced. Decreased falls among the elderly, improved access to prostheses for amputees, and a substantial decrease in the rate of complications from spinal cord injury were the resulting improvements of these measures.

#### 3.1.9. Registry, Research, and Auditing

There is a need to have an adequate registry of trauma as this remains a leading cause of morbidity and mortality in children across the world, and particularly in LMICs.

Amatoa et al. could clearly show that risk-adjusted pediatric trauma-related mortality is significantly lower in the US compared to India. Interestingly, they further found that the comparative odds of mortality are highest for children with lower injury and physiologic severity. Therefore, it could be assumed that simple targeted interventions such as standard timely trauma care, protocols, training, and early imaging could improve pediatric injury mortality in India [28].

Establishing a quality improvement program: Hashmi et al. reported on the direct assessment of the impact of establishing a quality improvement program in a tertiary hospital in Karachi, Pakistan. The study revealed a reduced mortality after implementation of trauma related quality improvement initiatives, i.e., morbidity and mortality meetings, a trauma quality improvement committee, and a trauma registry with regular audits [29].

Multiple injury severity scores have been used for trauma patient evaluation and studies in the developing world. One example is the Kampala Trauma Score (KTS), which was developed for use in resource-limited settings and has subsequently shown to be a robust predictor of death when compared to other trauma scoring systems [30].

#### 3.1.10. Specialization in Pediatric Trauma

Training for specialization in pediatric trauma is a long process, usually a five- or six-year residency for general surgery, followed by a year or two of surgical critical care/trauma fellowship. The course is essentially dedicated to the management of trauma in adults and may have a limited pediatric content. Trauma is therefore an established subspecialty of general surgery while the same does not already exist as a separate domain in pediatric surgery, except at a very limited scale and is essentially confined to HICs. Currently there is training and qualification in pediatric surgery not in trauma, which is a totally different approach so far. Improving training of attendants in pediatric trauma is a necessity and therefore it is recommended to address the following issues:

- Clear need to develop pediatric trauma as its own specialty (trauma fellowship/pediatric surgery);
- Educational and training institutions to adopt and introduce curricula and qualifications;
- Public health education;
- Medical legislations;
- Development of specialized trauma centers.

#### 3.1.11. Capacity and Confidence

There is a need for capacity building in pediatric trauma, specifically for dedicated pediatric trauma surgeons to deal with problems, particularly major trauma due to road traffic injuries (RTA), domestic injuries, armed conflicts, and burn injuries. Establishment of trauma systems including prehospital care and specialized trauma centers require adequate funding, infrastructural improvement, and personnel training. The outcome of more seriously and younger injured children is better when they are treated at a children’s hospital trauma center or at a trauma center that integrates pediatric and adult trauma services [23,31,32].

#### 3.1.12. Prevention and Initial Management

The development of injury prevention initiative specifically to individual countries should be a public health focus and a priority to reduce the burden of trauma injuries in LMICs [33]. Safety measure awareness can be delivered through schools and all available audio-visual media (newspapers, official, commercial, and social). Educational material, leaflets can be better utilized by making them available and easily accessible to the public including those in remote areas (Table 2). Delivery to the residences can be facilitated through nearby healthcare institutions, governmental or non-Governmental Organisation (NGO) bodies. It is imperative to know that 30% of pediatric trauma are avoidable. In this regard, GICS issued trauma prevention leaflets and they are available on the GICS website (https://www.globalchildrenssurgery.org/wp-content/uploads/2021/09/GICS-Trauma-handouts-Checklist.pdf, accessed 4 April 2022).

## 4. Discussion

Trauma remains a major cause of disability and death and disability. About 95% of the 5.8 million deaths each year, occur in LMICs. This includes approximately 1 million children. Additional deleterious effects have been seen on children, international organizations such as the United Nations by the increase of global conflicts [34].

Organized trauma care systems have been shown to save lives. Therefore, it is of outmost importance to introduce injury prevention strategies, resource-adapted trauma care protocols and specialized centers for trauma care in LMIC. These methods should facilitate better outcomes for critically ill and injured children.

It is crucial for the improvement of survival after major trauma to enhance the commitment of the LMIC governments to work alone and especially in collaboration with international non-governmental organizations (NGOs). This is to provide adequate healthcare to all people and should further improve survival after major trauma. Shanthakumar et al. reviewed forty-five studies and found that the main problems with trauma care in LMICs were education, operational measures, and infrastructure. Improvements in these areas would lead to a better healthcare system, which should result in a reduction of mortality in trauma-related injuries [35].

To date most trauma related research comes from HIC’s, but most injuries occur in LMICs.

Recently, Reynolds et al. studied about the possible impact of trauma care systems in LMICs and looked at 4284 records [36]. Only 71 reports from 32 countries met their inclusion criteria. They identified training, prehospital systems, and overall system organization as the most reported interventions. Quality-improvement, costing, rehabilitation, and legislation and governance were relatively neglected areas. Included reports may inform trauma care system planning in LMICs, and noted gaps may guide research and funding agendas.

With appreciation of the significance of pediatric trauma as a global health problem, and with consideration to all the reports and recommendations in the literature that address this issue, GICS seeks to propose the GICS children’s trauma care initiative based on the above mentioned 12 points as a practical framework for a comprehensive approach to the problem particularly in LMICs. It is needless to mention that there are many bodies, organizations, societies, and charities that work towards the same goals.

Examples of such organizations include: PTCF (Primary Trauma Care Foundation), PTS (Pediatric Trauma Society, USA), Child Safety Organizations, the Safe Kids Worldwide, ATLS (Advanced Trauma Life Support), WHO (Trauma Basic Emergency Care Course), and now, GICS initiative. Some of the courses are essentially designed for adults, with very limited pediatric content. Most of the activities of these organizations are directed to healthcare providers, but some of them are directed to the public. Those established organizations have vast wealth of experience, material and practical programs conducted on the ground. Tremendous efforts are undoubtedly exerted, and their input is clearly demonstrated in the clinical practice in HIC and, when accessible, LMIC, however, the activities are scattered and mostly in-coordinated.

The world has united previously to prevent potentially serious debilitating and fatal communicable diseases of childhood by immunization. It is time for the world to unite to face another potentially very serious, disabling, and killer childhood disease, that is pediatric trauma related injuries. In some HIC, pediatric trauma care as a specialty is completely established, in other places it is in infancy, whereas in the majority of LMIC it is still in conception. It is time for childhood care, welfare and protection societies and organizations to work in collaboration, direct their efforts and material to people in LMICs and ensure they benefit from those resources.

### To Achieve the GICS CTC Initiative Goals, the Following Steps Are Followed

1. GICS strives to promote the formulation of a global network of the existing, established societies dealing with pediatric trauma by invitation to GICS forum to exchange ideas, identify areas of mutual interest, and draw plans to achieve informed and efficient complementary cooperation and collaboration for a better distribution of resources.
2. The best way to achieve this is to encourage a group of pediatric surgeons to develop interest, enthusiasm, and ownership of the subject, which can be called a trauma ownership program/project (TOP); gather and build a team of surgeons and paramedics with motivation to run the service properly and to observe quality integrated and coordinated care, move towards specialization, development of a trauma curriculum and specialties, and eventually, establishment of specialized centers.
3. Public healthcare providers are important partners to be instructed and frequently provided with information about immediate and early management of trauma. Injured victims are initially handled by attending witnesses and audience who should equally, in fact more importantly, be provided with such information. Each broadcasted health educational program should dedicate a few minutes to teaching the public about methods of initial management of pediatric trauma.
4. GICS works towards acquiring the capacity to run training activities. In this regard, a one-day Children’s Primary Trauma Course is prepared to be conducted at the coming GICS physical meetings. There are many capable, well-established bodies with a vast wealth of experience in conducting well-structured training programs, access to which can be facilitated by GICS.
5. Furthermore, for advocacy, GICS invites healthcare professionals to voluntarily adopt, establish, and develop viable CTC programs locally and nationally according to their respective needs and available facilities to the best of their potentials and abilities.
6. For advanced trauma care at the hospital level, and for acquisition of appropriate and adequate facilities, healthcare responsible for managing children’s trauma should engage with the local authorities, child welfare organizations, and charities to plan and establish the required quality services. Standards for a skilled workforce, adequate equipment, infrastructure, and supplies, and documenting, monitoring, and evaluation of access to and quality of services, should be embedded in programs and legislation based on current knowledge and considerations promoting the right to the enjoyment of the highest attainable standard of health.

## 5. Conclusions

Pediatric trauma is a major health threat with significant socioeconomic implications. There is marked discrepancy and inequity in prehospital and hospital services between HICs and LMICs. GICS proposed a children’s trauma care initiative based on adopting a TOP: trauma ownership project/program to address pediatric trauma comprehensively utilizing available resources efficiently, and engaging and collaborating with concerned NGOs. There is a genuine need to draw attention of all stakeholders to the importance of trauma prevention, raising awareness, health education, proper training of HCW, encouragement of specialization in pediatric trauma, and allocation of funds to hospitals and all healthcare facilities that provide pediatric trauma services to achieve timely, efficient, and appropriate management of this non-discriminating, but potentially avoidable, devastating, disabling, disfiguring, greatly distressing, and expensive health problem.

GICS CTC initiative aims to improve pediatric trauma care in LMICs by developing injury prevention strategies; optimizing the use of locally available resources; obtaining commitment by LMIC governments; improvement in all fields of hospital care; improvements in infrastructure, education and training, and attention to data registry and research.

## Figures and Tables

**Table 1:** Trauma emergency tray checklist.

Items		Medications
- Ambu bag - face mask - Guedel oropharyngeal airway - cervical collar - laryngoscope with fresh batteries - endotracheal tubes/LMA different sizes - Magill forceps - suction machine, and tubes - a full oxygen cylinder (or central gas sockets) with an adapter - tracheostomy set - chest drains 8–24 Fr and underwater seal bottles - cannulae - CVL - intraosseous needle - IV fluids (Normal saline/Dextrose with different strengths—Ringer’s lactate - blood products	- cut down set and instruments (sterile towels and gowns sponge forceps, scalpel, tissue forceps toothed and non-toothed, needle holder, artery clips mosquitoes, hook, Ragnell retractors, tissue, and suture scissors) - syringes - local anesthesia vials - suture material (silk, vicryl) - antiseptic solutions - gauze swabs - adhesive plasters - feeding/nasogastric tubes - urinary catheters of different sizes - urine bag - gloves - cystofix set - thermometer	- Adrenaline - Hydrocortisone - Access to adequate analgesia - Calcium Gluconate - Sodium Bicarbonate - Frusemide - antibiotics - Tetanus antitoxin Devices - DC shock device - monitors - slabs - POP - specimen collection tubes and bottles - investigation request forms (lab and radiology)

**Table 2:** At a glance checklist: A practical approach to children’s trauma prevention.

Cause	Risk Factors	Action
Road Traffic Injuries (RTI)	Uneven roads	Pavements
	Reckless driving	Road signs, speed limit
	Vehicles safety	Legislations
	Seating and seat belts	Enforcement
	Carrying children	Legislations
	Bike size	Appropriate selection
	Bike safety	Brake, lights, bell
	Helmets	Enforcement
Falls	Climbing trees	Prevention, seasonal attention
	Incomplete buildings	Construction preventative measures
	Open windows	Grill windows
	Stairs	Safety gates
	Playgrounds	Soft ground, sand, watching
Burns	Scalds	Keep away, rotate pan handle.
	Fire	Avoid access to candles and cooking fire,
		Keep lighters out of reach.
		fireworks under supervision
		Avoid flammable clothes.
	Gas	Keep children away
Electrocution	Power sockets	Cover sockets Keep out of site
House utilities	Kitchen knives	Always keep out of reach of children
Drowning	Access to water pool,	Do not leave unwatched near pool.
	rivers, ponds,	Conduct swimming lessons.
	manholes	Cover
FB Ingestion	Loose batteries Magnets Sharp objects Chemical liquids	Manufacture safety measures, battery chamber security, inaccessibility. Careful disposal of used batteries Close observation Keep magnets away from children, ban selling. Keep objects, chemicals, and bleaches out of reach of children
Poisoning	Accessibility of medicines	Good, secured storage

## Data Availability

Not applicable.
